# Supplementary figures and images for: Diosgenin biosynthesis pathway and its regulation in Dioscorea cirrhosa L
Source: PeerJ. 2024 Jan 23;12:e16702. doi: 10.7717/peerj.16702 (PMC10812585; doi:10.7717/peerj.16702)

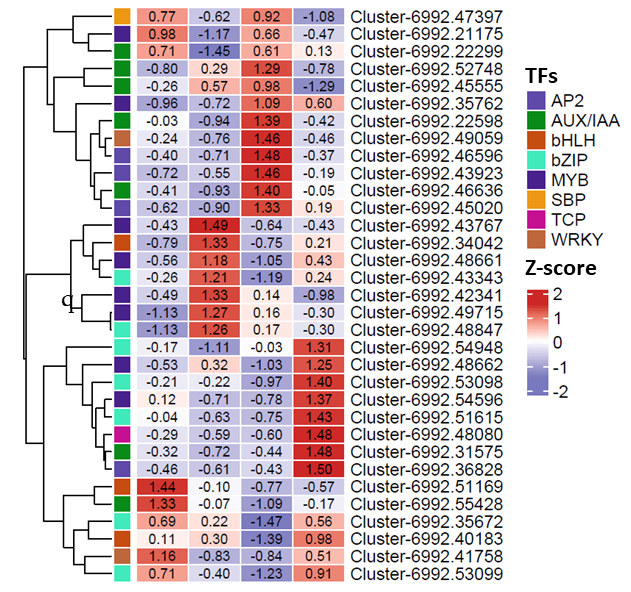

Supplement: Figure S1 — The color scale represents the average of FPKM value (scaled using Z-score), red color indicate high expression and blue color indicate low expression. [file peerj-12-16702-s001.png]
