# Supplementary material for: Diosgenin biosynthesis pathway and its regulation in Dioscorea cirrhosa L
Source: PeerJ. 2024 Jan 23;12:e16702. doi: 10.7717/peerj.16702 (PMC10812585; doi:10.7717/peerj.16702)
Supplement: Table S1 — Relative content of dioseginin metabolites. HPLC-MS/MS analyses were performed on three biological replicates. [file peerj-12-16702-s002.docx]

| **Compounds** | **LR** | **RD** | **DR** | **BR** |
| --- | --- | --- | --- | --- |
| Ruscogenin-1-*O*-xylosyl(1,3)fucoside | 0.00E+00 | 0.00E+00 | 0.00E+00 | 6.70E+04 |
| Diosgenin-3-*O*-rhamnosyl-(1→3)glcoside | 0.00E+00 | 0.00E+00 | 3.23E+03 | 1.06E+06 |
| Diosgenin-3-*O*-rhamnosyl(1,2)glcoside | 0.00E+00 | 0.00E+00 | 0.00E+00 | 1.52E+06 |
| Gracillin | 1.51E+06 | 6.35E+06 | 6.25E+06 | 2.26E+06 |
| Parisyunnanoside B | 2.72E+05 | 7.45E+04 | 1.26E+06 | 2.77E+05 |
| Diosgenin-3-*O*-glcosyl(1→4)rhamnosyl(1→4)rhamnosyl(1→2)glcoside | 1.72E+06 | 8.99E+05 | 3.36E+06 | 1.22E+06 |
| Pseudoprotodioscin | 2.61E+06 | 1.53E+06 | 4.89E+06 | 1.94E+06 |
| Protodioscin | 2.10E+04 | 1.02E+04 | 4.27E+04 | 2.90E+04 |
| Pennogenin-3-*O*-glucoside | 2.77E+04 | 6.85E+04 | 7.26E+04 | 8.02E+04 |
| Trillin-6'-*O*-glucoside | 9.96E+05 | 9.86E+05 | 3.64E+06 | 6.56E+05 |
| Trillin-6'-*O*-sophorotrioside | 1.04E+04 | 0.00E+00 | 1.73E+04 | 0.00E+00 |
| Trillin (Diosgenin-3-*O*-glucoside) | 2.03E+06 | 9.34E+05 | 2.69E+06 | 1.28E+06 |
| 3-*O*-(2-*O*-Acetyl-glucosyl)oleanolic acid | 1.76E+04 | 0.00E+00 | 1.36E+04 | 7.86E+03 |

**Table S1.** Diosgenin metabolites and their relative contents in *D. cirrhosa* tubers.

Note: Relative content of dioseginin metabolites. HPLC-MS/MS analyses were performed on three biological replicates.
