# Supplementary material for: Diosgenin biosynthesis pathway and its regulation in Dioscorea cirrhosa L
Source: PeerJ. 2024 Jan 23;12:e16702. doi: 10.7717/peerj.16702 (PMC10812585; doi:10.7717/peerj.16702)
Supplement: Table S2 [file peerj-12-16702-s003.docx]

**Table S2.** FPKM value of identified diosgenin biosynthesis pathway genes.

| **Gene** | **LR** | **RD** | **DR** | **BR** |
| --- | --- | --- | --- | --- |
| AACT | 2.203333333 | 6.883333333 | 2.726666667 | 1.65 |
| AACT | 20.45333333 | 12.37 | 14.01333333 | 12.79666667 |
| AACT | 58.51666667 | 39.04666667 | 82.44666667 | 29.81666667 |
| AACT | 265.2066667 | 274.1033333 | 163.1066667 | 157.9633333 |
| C14-R | 21.42333333 | 18.72333333 | 12.43 | 11.83666667 |
| C14-R | 64.53333333 | 58.95 | 28.25 | 35.83333333 |
| C5(6) | 21.07333333 | 36.26 | 18.97333333 | 8.036666667 |
| C5(6) | 31.79333333 | 13.82333333 | 5.3 | 0.966666667 |
| CAS | 22.87333333 | 22.61 | 11.59666667 | 19.46666667 |
| CPI | 27.82666667 | 33.45666667 | 47.28666667 | 7.21 |
| DXR | 29.01 | 28.10333333 | 28.30333333 | 18.33333333 |
| DXS | 24.11 | 13.6 | 64.52 | 14.12333333 |
| FDS | 68.15333333 | 78.18333333 | 49.87333333 | 34.60333333 |
| FDS | 34.35333333 | 53.12 | 33.85 | 27.07 |
| FDS | 24.58666667 | 10.90333333 | 7.333333333 | 18.21333333 |
| gcpE | 16.26333333 | 21.58 | 21.9 | 23.82666667 |
| GPS | 6.686666667 | 18.97666667 | 11.26333333 | 0.92 |
| HMGCR | 88.41333333 | 156.5433333 | 51.76333333 | 183.4433333 |
| HMGCR | 14.74 | 8.403333333 | 23.29 | 49.86 |
| HMGCR | 14.92 | 19.36666667 | 9.533333333 | 3.64 |
| HMGCR | 10.54 | 8.36 | 4.853333333 | 2.406666667 |
| HMGCR | 47.56666667 | 55.45 | 51.31666667 | 50.04333333 |
| HMGCR | 100.49 | 42.36333333 | 50.94333333 | 110.74 |
| HMGCR | 36.5 | 62.24333333 | 31.41333333 | 25.98 |
| HMGCS | 10.05666667 | 20.84 | 12.72666667 | 13.24333333 |
| IDI | 71.87 | 71.86666667 | 70.24 | 105.1533333 |
| IDI | 2272.976667 | 700.3666667 | 1606.373333 | 1082.336667 |
| ispD | 10.71666667 | 7.023333333 | 15.47666667 | 5.2 |
| ispE | 14.03666667 | 2.676666667 | 18.16666667 | 15.01333333 |
| ispE | 18.00333333 | 27.43333333 | 12.13666667 | 17.66 |
| ispF | 9.653333333 | 8.68 | 16.16333333 | 4.176666667 |
| ispH | 33.76333333 | 41.24 | 44.24666667 | 25.59666667 |
| MVD | 17.78 | 21.19666667 | 16.38 | 9.37 |
| MVK | 75.32666667 | 110.7966667 | 80.22 | 18.01333333 |
| MVK | 8.296666667 | 6.693333333 | 7.453333333 | 4.633333333 |
| MVK | 11.72333333 | 4.53 | 11.89 | 4.533333333 |
| SE | 35.47666667 | 52.56 | 17.56666667 | 51.77333333 |
| SMT1 | 9.44 | 14.37 | 14.14 | 10.49333333 |
| SMT1 | 15.39333333 | 12.21 | 18.14333333 | 13.9 |
| SMT1 | 13.24 | 12.89 | 0 | 0.776666667 |
| SS | 1.716666667 | 4.566666667 | 3.253333333 | 0.88 |
| SS | 8.79 | 17.46333333 | 6.713333333 | 5.123333333 |
| SS | 24.79 | 0.38 | 29.89333333 | 2.116666667 |
