# Supplementary material for: Diosgenin biosynthesis pathway and its regulation in Dioscorea cirrhosa L
Source: PeerJ. 2024 Jan 23;12:e16702. doi: 10.7717/peerj.16702 (PMC10812585; doi:10.7717/peerj.16702)
Supplement: Table S3 [file peerj-12-16702-s004.docx]

Table S3. Expression level of cadidate TFs (FPKM value).

| **TFs** | **LR** | **RD** | **DR** | **BR** |
| --- | --- | --- | --- | --- |
| WRKY | 312.01 | 135.0633333 | 545.4766667 | 522.92 |
| WRKY | 393.7233333 | 130.3733333 | 417.1633333 | 110.84 |
| WRKY | 1063.73 | 114.5033333 | 274.27 | 1381.18 |
| MYB | 39.64333333 | 12.96666667 | 10.95666667 | 27.35 |
| MYB | 65.96666667 | 12.66666667 | 9.68667 | 266.62 |
| MYB | 125.52 | 177.54 | 304.1133333 | 368.85 |
| MYB | 185.4466667 | 171.5433333 | 114.5066667 | 912.11 |
| MYB | 107.3966667 | 60.64 | 31.24 | 145.57 |
| MYB | 40.02333333 | 25.52333333 | 13.99 | 160.82 |
| MYB | 348.52 | 338.7633333 | 564.9066667 | 461.02 |
| MYB | 187.65 | 102.21 | 377.6266667 | 501.43 |
| MYB | 20.47333333 | 11.01666667 | 13.23 | 61.89 |
| MYB | 7.103333333 | 19.51 | 20.96223 | 104.77 |
| bZIP | 293.07 | 114.97 | 368.1966667 | 698.1 |
| bZIP | 87.67333333 | 46.00666667 | 11.27666667 | 468.36 |
| bZIP | 37.97666667 | 33.75666667 | 27.13 | 19.23 |
| bZIP | 276.7433333 | 89.99 | 191.56 | 925.44 |
| bZIP | 168.9966667 | 136.1966667 | 274.3133333 | 368.34 |
| bZIP | 239.1466667 | 243.45 | 305.1233333 | 356.22 |
| bZIP | 123.2966667 | 131.47 | 46.50333333 | 309.73 |
| bZIP | 200.4633333 | 84.86 | 57.08333333 | 345.02 |
| bZIP | 35.54666667 | 99.98333333 | 104.3266667 | 87.33 |
| bHLH | 32.73666667 | 19.87 | 20.5 | 117.46 |
| bHLH | 696.2566667 | 340.71 | 202.1667 | 565.05 |
| bHLH | 79.42333333 | 44.39 | 60.66333333 | 183.42 |
| bHLH | 11.41333333 | 13.24666667 | 25.28666667 | 35.88 |
| AUX/IAA | 235.2833333 | 133.9666667 | 360.13 | 422.57 |
| AUX/IAA | 36.95 | 9.993333333 | 16.60666667 | 62.27 |
| AUX/IAA | 217.8933333 | 143.66 | 390.45 | 243.3 |
| AUX/IAA | 2.033333333 | 3.923333333 | 12.86 | 0.53 |
| AP2/ERF | 32.4 | 12.27 | 37.14 | 113.42 |
| AP2/ERF | 7.003333333 | 21.99 | 96.76333333 | 18.39 |
| AP2/ERF | 7.773333333 | 18.48666667 | 51.86333333 | 21.68 |
| AP2/ERF | 110.1933333 | 3.463333333 | 945.56 | 223.61 |
| AP2/ERF | 278.88 | 52.22333333 | 1880.816667 | 1423.02 |
| AP2/ERF | 111.5833333 | 62.88666667 | 289.9166667 | 260.17 |
| AP2/ERF | 67.92 | 29.60333333 | 89.05666667 | 480.89 |
