# Supplementary material for: Diosgenin biosynthesis pathway and its regulation in Dioscorea cirrhosa L
Source: PeerJ. 2024 Jan 23;12:e16702. doi: 10.7717/peerj.16702 (PMC10812585; doi:10.7717/peerj.16702)
Supplement: Table S4 [file peerj-12-16702-s005.docx]

Table S4. Correlation analysis between TFs and diosgenin genes.

| **Gene** | **TFs** | r2 | **p** |
| --- | --- | --- | --- |
| SQS_3 | WRKY_47317 | 0.992359 | 0.007641 |
| MVK_2 | WRKY_47317 | 0.997937 | 0.002063 |
| HMGCR_5 | WRKY_47317 | -0.97231 | 0.027695 |
| HMGCR_4 | WRKY_47317 | -0.97231 | 0.027695 |
| SQS_1 | WRKY_41758 | -0.97435 | 0.025653 |
| HMGCR_7 | WRKY_41758 | 0.995744 | 0.004256 |
| SMT1_1 | MYB_68709 | -0.97289 | 0.027109 |
| FPS_3 | MYB_68709 | 0.991127 | 0.008873 |
| IDI_1 | MYB_54596 | 0.981272 | 0.018728 |
| DXR | MYB_54596 | -0.95843 | 0.041572 |
| HMGCR_1 | MYB_49715 | -0.9973 | 0.002697 |
| C14(R__1 | MYB_49715 | -0.98184 | 0.018161 |
| IDI_1 | MYB_48662 | 0.999298 | 0.000702 |
| DXR | MYB_48662 | -0.99083 | 0.009172 |
| ispH | MYB_44833 | -0.99161 | 0.008391 |
| IDI_1 | MYB_43761 | 0.99255 | 0.00745 |
| DXR | MYB_43761 | -0.97536 | 0.024636 |
| CAS | MYB_42341 | -0.96776 | 0.032235 |
| C14(R)_1 | MYB_42341 | -0.95976 | 0.04024 |
| HMGCR_2 | MYB_35762 | -0.99351 | 0.006485 |
| FPS_1 | MYB_35762 | -0.99876 | 0.001242 |
| AACT_4 | MYB_35762 | -0.95888 | 0.041117 |
| MVK_3 | MYB_34768 | -0.95371 | 0.046294 |
| IDI_1 | MYB_34768 | 0.986797 | 0.013203 |
| DXR | MYB_34768 | -0.96969 | 0.030305 |
| IDI_1 | MYB_33730 | 0.985684 | 0.014316 |
| DXR | MYB_33730 | -0.99756 | 0.002443 |
| MVK_3 | bZIP_54948 | -0.98408 | 0.015919 |
| MVD | bZIP_54948 | -0.99951 | 0.00049 |
| HMGCR_3 | bZIP_54948 | 0.986815 | 0.013185 |
| HMGCR_2 | bZIP_54948 | -0.97298 | 0.027025 |
| FPS_1 | bZIP_54948 | -0.95949 | 0.040512 |
| C5(6)_1 | bZIP_54948 | -0.95786 | 0.042143 |
| IDI_1 | bZIP_53098 | 0.993705 | 0.006295 |
| DXR | bZIP_53098 | -0.9775 | 0.022505 |
| HMGCR_1 | bZIP_51628 | 0.991839 | 0.008161 |
| MVK_3 | bZIP_51615 | -0.97238 | 0.027619 |
| MVD | bZIP_51615 | -0.95427 | 0.045733 |
| IDI_1 | bZIP_51615 | 0.977881 | 0.022119 |
| HMGCR_3 | bZIP_51615 | 0.954701 | 0.045299 |
| DXR | bZIP_51615 | -0.962 | 0.037997 |
| MVD | bZIP_48847 | -0.95372 | 0.046275 |
| HMGCR_3 | bZIP_48847 | 0.964563 | 0.035437 |
| HMGCR_2 | bZIP_48847 | -0.98978 | 0.010217 |
| FPS_1 | bZIP_48847 | -0.99555 | 0.004452 |
| HMGCR_3 | bZIP_47573 | 0.951615 | 0.048385 |
| HMGCR_2 | bZIP_47573 | -0.95476 | 0.045235 |
| HMGCR_1 | bZIP_47573 | -0.97214 | 0.027858 |
| FPS_1 | bZIP_47573 | -0.96906 | 0.030943 |
| IDI_1 | bZIP_43343 | 0.95371 | 0.04629 |
| ispH | bZIP_35672 | -0.99676 | 0.003236 |
| IDI_1 | bHLH_51919 | 0.993634 | 0.006366 |
| DXR | bHLH_51919 | -0.97958 | 0.020424 |
| SMT1_1 | bHLH_51169 | -0.9535 | 0.046497 |
| FPS_3 | bHLH_51169 | 0.993078 | 0.006922 |
| MVK_3 | bHLH_40183 | -0.97388 | 0.026116 |
| MVD | bHLH_40183 | -0.95059 | 0.049406 |
| IDI_1 | bHLH_40183 | 0.972966 | 0.027034 |
| DXR | bHLH_40183 | -0.95382 | 0.046175 |
| HMGCR_1 | bHLH_30724 | -0.97842 | 0.021579 |
| FPS_1 | bHLH_30724 | -0.95999 | 0.040013 |
| HMGCR_2 | AUX/IAA_52748 | -0.98593 | 0.014075 |
| FPS_1 | AUX/IAA_52748 | -0.98628 | 0.013716 |
| SQS_1 | AUX/IAA_46788 | -0.95028 | 0.049715 |
| MVK_3 | AUX/IAA_46788 | -0.95158 | 0.048421 |
| ispH | AUX/IAA_46788 | -0.96481 | 0.035187 |
| MVK_3 | AP2/ERF-ERF_54001 | -0.98057 | 0.01943 |
| MVD | AP2/ERF-ERF_54001 | -0.98437 | 0.015626 |
| IDI_1 | AP2/ERF-ERF_54001 | 0.961966 | 0.038034 |
| HMGCR_3 | AP2/ERF-ERF_54001 | 0.988074 | 0.011926 |
| DXR | AP2/ERF-ERF_54001 | -0.95745 | 0.04255 |
| CAS | AP2/ERF-ERF_50781 | -0.96025 | 0.039753 |
| CAS | AP2/ERF-ERF_48262 | -0.9745 | 0.025495 |
| DXS | AP2/ERF-ERF_46596 | 0.958767 | 0.041233 |
| CAS | AP2/ERF-ERF_46596 | -0.98629 | 0.013708 |
| SMT1_3 | AP2/ERF-ERF_45020 | -0.97774 | 0.022263 |
| C14(R)_1 | AP2/ERF-ERF_45020 | -0.97187 | 0.028129 |
| AACT_4 | AP2/ERF-ERF_45020 | -0.96856 | 0.03144 |
| SMT1_3 | AP2/ERF-ERF_43923 | -0.97783 | 0.022169 |
| C14(R)_1 | AP2/ERF-ERF_43923 | -0.95005 | 0.049954 |
| AACT_4 | AP2/ERF-ERF_43923 | -0.9822 | 0.017801 |
| MVD | AP2/ERF-ERF_36828 | -0.95459 | 0.045407 |
| IDI_1 | AP2/ERF-ERF_36828 | 0.988062 | 0.011938 |
| HMGCR_3 | AP2/ERF-ERF_36828 | 0.973803 | 0.026197 |
| DXR | AP2/ERF-ERF_36828 | -0.98697 | 0.013033 |
