# Supplementary material for: Diosgenin biosynthesis pathway and its regulation in Dioscorea cirrhosa L
Source: PeerJ. 2024 Jan 23;12:e16702. doi: 10.7717/peerj.16702 (PMC10812585; doi:10.7717/peerj.16702)
Supplement: Table S5 [file peerj-12-16702-s006.docx]

**Table S5.** Annotation information of candidate CYP450 genes.

| **ID** | **KEGG ID** | **EC** | **CYP450** | **Species** |
| --- | --- | --- | --- | --- |
| Cluster-6992.40273 | K07408 | EC:1.14.14.1 | CYP450 71A1 | *Elaeis guineensis* |
| Cluster-6992.42754 | K07408 | EC:1.14.14.1 | CYP450 89A2 | *Elaeis guineensis* |
| Cluster-6992.42926 | K10717 |  | CYP450 313a4 | *Elaeis guineensis* |
| Cluster-6992.43136 | K20617 |  | CYP450 71A1 | *Elaeis guineensis* |
| Cluster-6992.43378 | K20617 |  | CYP450 71A1 | *Elaeis guineensis* |
| Cluster-6992.43385 | K20617 |  | CYP450 71A1 | *Elaeis guineensis* |
| Cluster-6992.45560 | K20617 |  | CYP450 71A1 | *Elaeis guineensis* |
| Cluster-6992.46280 | K20617 |  | CYP450 71A1 | *Elaeis guineensis* |
| Cluster-6992.47541 | K20617 |  | CYP450 71A1 | *Elaeis guineensis* |
| Cluster-6992.51902 | K20617 |  | CYP450 71A1 | *Elaeis guineensis* |
| Cluster-6992.5341 | K20495 | EC:1.14.14.80 | CYP450 704C1 | *Elaeis guineensis* |
| Cluster-6992.54954 | K20665 | EC:1.14.14.48 | CYP450 94A1 | *Elaeis guineensis* |
| Cluster-6992.59287 | K09588 | EC:1.14.-.- | CYP450 90A1 | *Elaeis guineensis* |
| Cluster-6992.59734 | K07426 | EC:1.14.14.1 | CYP450 72A219 | *Elaeis guineensis* |
| Cluster-6992.59979 | K07418 | EC:1.14.14.1 | CYP450 71A1 | *Elaeis guineensis* |
| Cluster-6992.63215 | K07426 | EC:1.14.14.1 | CYP450 72A219 | *Elaeis guineensis* |
| Cluster-6992.65040 | K07409 | EC:1.14.14.1 | CYP450 89A2 | *Elaeis guineensis* |
| Cluster-6992.517 | K07418 | EC:1.14.14.1 | CYP450 71A1 | Musa acuminata subsp. malaccensis |
| Cluster-6992.66292 | K20771 | EC:1.14.-.- | CYP450 711A1 | Musa acuminata subsp. malaccensis |
| Cluster-6992.33753 | K05917 | EC：1.14.13.70 | CYP450 51 | *Asparagus officinalis* |
| Cluster-6992.33754 | K05917 | EC：1.14.13.70 | CYP450 51 | *Asparagus officinalis* |
| Cluster-6992.39991 | K21693 | EC:1.14.14.98 | CYP450 82D47 | *Asparagus officinalis* |
| Cluster-6992.66603 | K07426 | EC:1.14.14.1 | CYP450 72A219 | Daucus carota subsp. sativus |
| Cluster-6992.33292 | K09587 | EC:1.14.13.- | CYP450 90B1 | *Phoenix dactylifera* |
| Cluster-6992.40454 | K13260 | EC:1.14.13.89 1.14.13.53 | CYP450 81E8 | *Phoenix dactylifera* |
| Cluster-6992.41463 | K15402 | EC:1.14.-.- | CYP450 94A1 | *Phoenix dactylifera* |
| Cluster-6992.43387 | K20617 |  | CYP450 71A1 | *Phoenix dactylifera* |
| Cluster-6992.47040 | K20617 |  | CYP450 71A1 | *Phoenix dactylifera* |
| Cluster-6992.48174 | K09587 | EC:1.14.13.- | CYP450 90B1 | *Phoenix dactylifera* |
| Cluster-6992.50836 | K20771 | EC:1.14.-.- | CYP450 711A1 | *Phoenix dactylifera* |
| Cluster-6992.59090 | K07426 | EC:1.14.14.1 | CYP450 72A219 | *Phoenix dactylifera* |
| Cluster-6992.61084 | K15639 | EC:1.14.-.- | CYP450 734A1 | *Phoenix dactylifera* |
| Cluster-6992.66042 | K07426 | EC:1.14.14.1 | CYP450 72A219 | *Phoenix dactylifera* |
| Cluster-6992.63413 | K09588 | EC:1.14.-.- | CYP450 90A1 | *Ananas comosus* |
| Cluster-6992.31785 | K20617 |  | CYP450 71A1 | *Phoenix dactylifera* |
| Cluster-6992.67001 | K20623 |  | CYP450 71A1 | *Phoenix dactylifera* |
| Cluster-6992.31789 | K20771 | EC:1.14.-.- | CYP450 711A1 | Musa acuminata subsp. malaccensis |
| Cluster-6992.77886 | K20624 | EC:1.14.14.49 | CYP450 94C1 | Musa acuminata subsp. malaccensis |
| Cluster-6992.51327 | K00327 | EC:1.6.2.4 | NADPH-CYP450 | *Elaeis guineensis* |
| Cluster-6992.66001 | K20624 | EC:1.14.14.49 | CYP450 94C1 | *Elaeis guineensis* |
