# Supplementary material for: Diosgenin biosynthesis pathway and its regulation in Dioscorea cirrhosa L
Source: PeerJ. 2024 Jan 23;12:e16702. doi: 10.7717/peerj.16702 (PMC10812585; doi:10.7717/peerj.16702)
Supplement: Table S6 [file peerj-12-16702-s007.docx]

**Table S6.** Correlation analysis between CYP450 gene and diosgenin metabolites.

| **Gene** | **Metabolite** | r2 | **p** |
| --- | --- | --- | --- |
| Cluster-6992.63413 | Trillin-6'-O-glucoside | 0.993377597 | 0.006622403 |
| Cluster-6992.54954 | Trillin-6'-O-glucoside | 0.992723766 | 0.007276234 |
| Cluster-6992.46280 | Trillin-6'-O-glucoside | 0.989950911 | 0.010049089 |
| Cluster-6992.63215 | Trillin-6'-O-glucoside | 0.987790978 | 0.012209022 |
| Cluster-6992.51902 | Trillin-6'-O-glucoside | 0.979957224 | 0.020042776 |
| Cluster-6992.41463 | Trillin-6'-O-glucoside | 0.975896912 | 0.024103088 |
| Cluster-6992.66001 | Trillin-6'-O-glucoside | 0.964601714 | 0.035398286 |
| Cluster-6992.43136 | Trillin-6'-O-glucoside | 0.962188738 | 0.037811262 |
| Cluster-6992.66042 | Trillin-6'-O-sophorotrioside | 0.992387622 | 0.007612378 |
| Cluster-6992.517 | Trillin-6'-O-sophorotrioside | 0.990651396 | 0.009348604 |
| Cluster-6992.67001 | Trillin-6'-O-sophorotrioside | 0.988592943 | 0.011407057 |
| Cluster-6992.40454 | Trillin-6'-O-sophorotrioside | 0.971261507 | 0.028738493 |
| Cluster-6992.45560 | Trillin-6'-O-sophorotrioside | 0.964143592 | 0.035856408 |
| Cluster-6992.66001 | Trillin-6'-O-sophorotrioside | 0.953420751 | 0.046579249 |
| Cluster-6992.59287 | Trillin-6'-O-sophorotrioside | 0.951486411 | 0.048513589 |
| Cluster-6992.41463 | PaParisyunnanoside B | 0.998646064 | 0.001353936 |
| Cluster-6992.54954 | PaParisyunnanoside B | 0.985595923 | 0.014404077 |
| Cluster-6992.66001 | PaParisyunnanoside B | 0.962738124 | 0.037261876 |
| Cluster-6992.63413 | PaParisyunnanoside B | 0.959565459 | 0.040434541 |
| Cluster-6992.66001 | Pseudoprotodioscin | 0.992918434 | 0.007081566 |
| Cluster-6992.41463 | Pseudoprotodioscin | 0.987144839 | 0.012855161 |
| Cluster-6992.517 | Pseudoprotodioscin | 0.976479121 | 0.023520879 |
| Cluster-6992.63413 | Pseudoprotodioscin | 0.968482251 | 0.031517749 |
| Cluster-6992.54954 | Pseudoprotodioscin | 0.955956115 | 0.044043885 |
| Cluster-6992.66001 | Diosgenin-3-O-glcosyl(1→4)rhamnosyl(1→4)rhamnosyl(1→2)glcoside | 0.992186192 | 0.007813808 |
| Cluster-6992.41463 | Diosgenin-3-O-glcosyl(1→4)rhamnosyl(1→4)rhamnosyl(1→2)glcoside | 0.985455601 | 0.014544399 |
| Cluster-6992.517 | Diosgenin-3-O-glcosyl(1→4)rhamnosyl(1→4)rhamnosyl(1→2)glcoside | 0.978085668 | 0.021914332 |
| Cluster-6992.63413 | Diosgenin-3-O-glcosyl(1→4)rhamnosyl(1→4)rhamnosyl(1→2)glcoside | 0.965453935 | 0.034546065 |
| Cluster-6992.54954 | Diosgenin-3-O-glcosyl(1→4)rhamnosyl(1→4)rhamnosyl(1→2)glcoside | 0.952220844 | 0.047779156 |
| Cluster-6992.67001 | Trillin (Diosgenin-3-O-glucoside) | 0.996617539 | 0.003382461 |
| Cluster-6992.517 | Trillin (Diosgenin-3-O-glucoside) | 0.986358687 | 0.013641313 |
| Cluster-6992.66042 | Trillin (Diosgenin-3-O-glucoside) | 0.971778269 | 0.028221731 |
| Cluster-6992.40273 | Trillin (Diosgenin-3-O-glucoside) | 0.952218944 | 0.047781056 |
| Cluster-6992.59734 | Pennogenin-3-O-glucoside | -0.962458232 | 0.037541768 |
| Cluster-6992.40273 | 3-O-(2-O-Acetyl-glucosyl)oleanolic acid | 0.952044043 | 0.047955957 |
